# Sovereignty in the Digital and Contact Tracing Apps

**DOI:** 10.1007/s44206-022-00030-2

**Published:** 2022-12-26

**Authors:** Max Tretter

**Affiliations:** Department of Theology, Friedrich-Alexander-Universität Erlangen-Nürnberg, Erlangen, Bavaria Germany

**Keywords:** Sovereignty, Digital sovereignty, Contact tracing apps, COVID-19, Data sovereignty, Digital governance

## Abstract

Recently, the concept of sovereignty in the digital has attracted much attention. Several publications dealing with this concept assume that it can best be described as a network of different, overlapping exercises of power. Nevertheless, there is a need for further research on how exactly sovereignty in the digital can be understood. In order to contribute to a better understanding of this concept, I illustrate its complex structure using contact tracing apps as a paradigmatic example. I conduct a narrative review to show what sovereignty looks like in the context of these apps. In the context of digital contact tracing apps, sovereignty is best understood as a complex network of three actors—nations, (big tech) companies, and individuals—that exercise various forms of power against or on behalf of each other to claim sovereignty for themselves and to either weaken or strengthen the sovereignty claims of other actors. Since large parts of the results can be generalized from the particular context of contact tracing apps, they contribute to a better overall understanding of the concept of sovereignty in digital. This might, in turn, be helpful for discussions about this technology as well as about the regulation and governance of the digital in general.

## Introduction

Recently, the concept of sovereignty in the digital has attracted much attention (Glasze et al., 2022b; Pohle, 2021). Not least in light of the ever-growing power of digital platforms (Bendig et al., 2019), the steady progress of artificial intelligence (Schneider, 2020) and new forms of digital warfare (Singer & Friedman, 2014), achieving sovereignty in the digital has been adopted by nations as a state goal (Klenk et al., 2020; Pohle & Thiel, 2021), by (tech) companies as a central guideline (D’Elia, 2016; Pohle & Thiel, 2020), and by individuals (Cardullo & Kitchin, 2018; Pohle, 2020b) or interest groups (Cooper, 2019; Stewart, 2017) as a directive.^1^

The more the “hype” (Obendiek, 2021) about this concept became, the more publications started addressing sovereignty in the digital as well. Publications with a more analytical focus examine which different actors are striving for sovereignty in the digital and what goals they are pursuing (Adonis, 2019; Hummel et al., 2021), or they focus on specific states or unions and examine their “digital sovereignty strategies” (Baischew et al., 2020). Publications with a more normative focus compare different strategies with each other (Schneider, 2020), evaluate them ethically (Pierri & Herlo, 2021), and some even make recommendations on how sovereignty in the digital should be pursued (Floridi, 2020; Pohle & Thiel, 2020). Publications with a more conceptual focus, meanwhile, attempt to understand the structure of this “complex concept” (Fabiano, 2020), i.e., explore how different actors interact with each other in the digital, how they strive for sovereignty, and how they influence each other in the process (Floridi, 2020; Glasze et al., 2022a; Pohle & Thiel, 2020).

The latter publications in particular have provided valuable insights into the structure of sovereignty in the digital. For example, Luciano Floridi suggests imagining “digital sovereignty” as a complex network of different actors exercising power against each other and trying to establish themselves as sovereigns (Floridi, 2020). Precisely because this proposal to think of sovereignty in the digital as a network—a proposal that is also put forward or implied in a similar way by other authors (Couture & Toupin, 2019; Hummel et al., 2021; Ruohonen, 2021)—seems so promising, it would be important to conduct further research at this point, to examine sovereignty networks in more detail and to work out how different actors exercise their power in the digital. This is where my study comes in. Using the example of contact tracing apps, which are especially used during the COVID-19 pandemic, it aims to analyze these network-like structures of sovereignty in the digital in more detail, to work out how different actors exercise their power during the development, implementation, and use of these apps to gain sovereignty—and thus to contribute to a better understanding of this concept. Consequently, the research question of my paper is how can we understand sovereignty in the context of digital contact tracing apps? And what conclusions does this allow about the structure of sovereignty in the digital? Ultimately, I aim to show that sovereignty in the context of digital contact tracing apps can be understood as a network of three actors—nations, (big tech) companies, and individuals—exerting their power both *against* and *for* each other to claim sovereignty for themselves and to weaken or strengthen others’ claims to sovereignty—and that these findings can be generalized and allow us to draw conclusions about sovereignty in the digital more generally.

To prove my point, I will examine how sovereignty is represented in the discussions around digital contact tracing by means of a narrative review. To provide some context and a preliminary understanding, I will first make some preliminary considerations about sovereignty in the analog and in the digital. Then I will explain why digital contact tracing apps are a good example to ask how to understand sovereignty in the digital. Afterwards I will lay out my method of narrative review before presenting my results—i.e., showing which actors play a central role in the discussions on contact tracing apps and how their exercises of power and claims for sovereignty are represented—and summarizing them in a discussion, bringing them into conversation with existing conceptions of “digital sovereignty,” and drawing final conclusions about the structure of sovereignty in the digital.

## The Complexity of the Concept of Sovereignty

In order to show the complexity of the concept of sovereignty, I will follow three steps. First, I will examine the basic structure of the concept of sovereignty. Then, I will describe what challenges a sovereign faces when he or she is confronted with other forms of power or with other sovereigns. Finally, I will reflect on sovereignty in the digital and show what challenges arise when transferring the concept of sovereignty to the digital. As a result of these preliminary considerations, I will show that sovereignty can best be understood as the “supreme authority within a territory” (Philpott, 2003). In the digital, however, there is no sovereignty in this sense. Instead, it is possible to observe how different actors exercise power in order to claim sovereignty, or to weaken or strengthen the sovereignty claims of others—and subsequently to describe *sovereignty in the digital as a complex network of exercises of power and claims to sovereignty*.

### The Basic Structure of Sovereignty

Sovereignty is related to the exercise of power (Bodin, 2010). Most fundamentally, sovereignty can be described as an *authority* that distinguishes legitimate exercises of power from illegitimate ones (Philpott, 1995). This can be illustrated by an example. For instance, it is an illegitimate exercise of power when robbers assault a person, but a legitimate exercise of power when police officers arrest those robbers—and it is an authority that determines that the arrest is legitimate but the assault is not.

However, such authority is never universal. For example, French authorities cannot simply send police officers to another country to arrest robbers there. Rather, it is the latter country’s authorities that must decide on the legitimacy of exercises of power on its territory. Ultimately, all authority is bound and limited to a territory. And if we understand sovereignty as authority, this means that sovereignty is also bound and limited to a territory (Stilz, 2019)*.*

Yet, it is usually the case that there are various authorities in an area that can decide on the legitimacy of the exercise of power. In the case of the police, this includes the legislator. After all, the police are only allowed to arrest people who break the law. Further, there is the chief of police, who can order police officers where to patrol and what crimes to focus on. Finally, individuals or companies can decide, for example, whether and to what extent to cooperate with law enforcement, what information to share, or to what extent to insist on privacy protections. While all of these actors have some sort of authority and can have a say in the legitimacy of the police’s exercise of power, not all of them are sovereigns. Rather, sovereignty refers exclusively to the *highest form of authority*, meaning that only that actor is sovereign who can decide on the authority of other actors, and the legitimacy of their power and, if necessary, also restrict it (Agamben, 1999; Schmitt, 2005). While in principle any actor can become sovereign, currently in most countries the state and its government hold supreme authority (Hobbes, 1997).

Summarizing these considerations, sovereignty can be described as the (1) supreme (2) authority (3) within a territory (Philpott, 2003).

### Other Sovereigns and Other Challenges to Sovereignty

If sovereignty describes the supreme authority within a territory, the question arises how it is affected by other forms of power or by other sovereigns. How, for example, does it affect the sovereignty of the state when other actors interfere with its authority? Or how do interactions with other sovereigns affect its own sovereignty?

On the one hand, it restricts a state’s sovereignty if it concludes treaties with other sovereign states or even joins forces with them, e.g., to form the European Union (EU) or NATO. This is because these treaties and alliances impose certain obligations on the state. For example, it must henceforth recognize the EU laws that are decided in Brussels and implement them in its territory, or it must spend a certain percentage of its gross domestic product on armaments and send soldiers on joint NATO missions. This means that in matters of legislation, finance and armaments, although the state has the supreme authority, it nevertheless cannot decide entirely on its own. Its sovereignty always proves limited and conditional in reality—and absolute sovereignty exists only on the drawing board. Similarly, a state’s sovereignty is limited to some extent by business lobbyists or nongovernmental organizations that influence legislation, or by democratization processes that limit the power and duration of governments. Likewise, the sovereignty of a state is limited to some extent by economic lobbyists (Woodley, 2015) or nongovernmental organizations (Sassen, 1996) that influence its legislation, or by democratization processes that limit the power and duration of governments (Brown, 2010).

On the other hand, it is also beneficial for the state and its sovereignty to enter into treaties with other states and join forces with them. As, for example, the political scientist Stephen Krasner shows in his research on sovereignty (Krasner, 1999, 2001), treaties or alliances can increase the international as well as national recognition of a state or government, and thus increase its legitimacy and strengthen its sovereignty. Likewise, being democratically constituted and having its government elected by the *demos*, as well as having a strong civil society, e.g., NGOs, and a thriving economy with successful businesses, can increase a state’s legitimacy, financial strength, agency, and sovereignty (Philpott, 2001).

While, in summary, there is only one sovereign per territory, usually the state and its government, there are many other actors exercising power in its territory, as well as sovereigns of other territories it interacts with. These interactions and other exercises of power have a dual effect on state sovereignty—they can both weaken and strengthen it.

### Sovereignty and the Digital

If the previous considerations show how complex sovereignty is in the analog, it becomes even more complex when thinking about sovereignty in the digital. There are two reasons for this. First, the complex relationships between the digital and the analog. Second, the fact that there is not yet a “supreme authority” in the digital. Both reasons are interrelated in many ways.

The relations between the digital and the analog are ambiguous (Glasze et al., 2022a). On the one hand, the digital is a separate territory. This can be seen, for example, in the fact that people from two completely different territories, some of which are even enemies in the analog world, can digitally communicate with each other in real time or that it is possible to call up information on the internet that was produced at the other end of the world. On the other hand, it is not independent of what can henceforth be called “analog” (Martin et al., 2022; Möllers, 2020). For without analog cables, servers, satellites, and the like, the digital simply could not exist (Martin et al., 2022). But not only is the digital dependent on the analog—conversely, the analog is also dependent on the digital. For example, many everyday technologies and structures would not work without the digital, e.g., the power grid, the transport network, global communications, and the financial system, since they are all digitally controlled or managed (Daucé & Musiani, 2021). To describe this interdependence and inseparability, digital philosopher Luciano Floridi coined the term “onlife” (Floridi, 2014).

These complex and ambiguous relations between the analog and the digital are the key reason why no one has been able to prove itself as the supreme authority in the digital. Instead, there are several actors who claim sovereignty in the digital realm (Adonis, 2019; Hummel et al., 2021; Schneider, 2020). First, there are digital companies as Microsoft, Google, Meta, and Tesla. They own large parts of the global digital infrastructure, i.e., cables, servers, satellites and the like, without which the digital would not exist. Since they own the relevant “hardware” and have the power to massively restrict or even shut down the digital by prohibiting the use of their infrastructure, they also claim to have supreme authority in the digital. Second, states also claim sovereignty in the digital. For although the digital forms a kind of territory of its own, it still requires a material infrastructure that is located in analog territories. States take this as an opportunity to extend their analog sovereignty to the digital as well—or at least to claim such sovereignty in the digital. Third, individuals also claim sovereignty in the digital. They justify their claim by pointing out that their data are the “raw material” of the digital, filling it with content and making it useful and valuable. To illustrate this, think for example of *Instagram* without user photos or *YouTube* without the clips of its users. As it is “their” data that circulates in the digital, however, individuals claim sovereignty over this data in particular and, as a result, over the digital in general.

Theoretically, sovereignty in the digital could also be described as the supreme authority in the digital. De facto, however, it is again more accurate to describe this sovereignty as the greatest—though never supreme—authority in the digital. Nevertheless, it remains unclear who holds the most authority. There are three actors who claim sovereignty in the digital, justify their claims differently, and attempt to gain sovereignty by exercising power as well as weakening the sovereignty claims of the other actors (Tretter, 2022a). But despite their various attempts, none of them can fully prevail over the others and become the supreme authority in the digital. This leads to the conclusion that, based on the above criteria, *there currently is no sovereignty in the digital*, but only various exercises of power through which sovereignty is claimed and the claims of others are attacked—and that when we speak of sovereignty in the digital, we are most likely describing this *network of various exercises of power that are connected to different claims of sovereignty* (Floridi, 2020).

## Contact Tracing Apps and Why They are Useful Examples for Analyzing Sovereignty in the Digital

Since this article aims to analyze the structure of sovereignty in the digital using the example of contact tracing apps—their development, implementation, and use during the COVID-19 pandemic—I will now present why exactly these apps are a good example to think about sovereignty and its network-like structures.

These apps work by collecting and combining two sets of data: first, their users’ contacts (Ahmad et al., 2021), which are either automatically collected through Bluetooth data exchange between smart devices (Stanley & Stisa Granick, 2020) or identified by health authorities, based on their users’ location data (Ahmad et al., 2021; Stanley & Stisa Granick, 2020); second, their users’ COVID-19 infection status. Based on these data, the infection risk of a user can be determined—if a user had no contact with a person who was tested COVID-19-positive, their risk of infection is calculated as low; if they had contact with one or more COVID-19-positive individuals, their estimated risk of infection increases. By tracking contacts, digital contact tracing apps can provide their users information about their own risk of infection or recommend them, if necessary, several preventive actions—such as getting tested for COVID-19 or going into voluntary self-quarantine—and can provide public health authorities insights into the spread of infection in society and provide them information about the course of the pandemic. The goal is to use contact tracing apps to detect and break infection chains and contribute to containing the spread of the virus, in the least invasive and most solidary way possible (Tretter, 2022b), and stopping the COVID-19 pandemic in the long term (Ferretti et al., 2020).

Contact tracing apps are a particularly good illustration of the complex entanglements of sovereignty in the digital as there are different actors involved with the development, implementation, and use of contact tracing apps, that—as we will see below—sometimes have different ideas about how to deal with the data produced. For example, should it only be accessible to users, to public health authorities in anonymized form, or without anonymization? To enforce their ideas, some actors exercise a great deal of power and try to influence or control other actors. These attempts to enforce their ideas and become the defining authority in the context of contact tracing apps are described by many scholars using the concept of (digital) sovereignty.

Given the complex interconnections and the various ways in which different actors seek to exercise power, it seems promising to consider contact-tracking apps from a sovereignty perspective—just as it can be productive, conversely, to perceive the various exercises of power as exemplifying struggles for sovereignty in the digital.

## Methods

To approach the question of how to understand sovereignty in the context of digital contact tracing apps, I will conduct a narrative review and elaborate how sovereignty is understood in the academic discourse on contact tracing apps (Dixon-Woods et al., 2005; Galvan & Galvan, 2017). The goal of this review is to first identify which actors are most frequently mentioned as “sovereigns” in the context of contact tracing apps during the COVID-19 pandemic and to sketch out how they are portrayed to articulate their sovereignty against each other.

I have chosen to focus on publications *about* contact tracing apps rather than on statements by the actors involved themselves, since “sovereignty” is primarily used as an interpretive concept by scholars. The actors involved themselves rarely use the term sovereignty when describing their decisions and actions in the context of these apps, instead referring to other concepts such as privacy, autonomy, governance. At most, nations occasionally use the term to describe their decisions and actions—but even they rarely do so. Nevertheless, the concept of sovereignty can help to understand how and why actors act the way they do in the context of these apps. This perspective of sovereignty is more prominent in analytical publications *about* contact tracing apps, which is why I have chosen to focus on it.

The first step was to create a sample of publications that use the concept of sovereignty to describe how different actors interact with each other in the context of contact tracing apps. This sample was intended to represent all uses of sovereignty in this context and therefore be as comprehensive as possible. In order to achieve this comprehensiveness and representativeness of the sample, I searched the databases *GIFT*, *Scopus*, *Web of Science*, and *Google Scholar* using the keyword combination “tracing apps” and “sovereignty” (see Table 1). Since the discussions around contact tracing apps has gained momentum with the COVID-19 pandemic I set January 01, 2020 as the start date for my sample. I completed the sampling process on February 17, 2021.

**Table 1:** Overview of the search, listing the names of the databases, the search strategies used, and the number of results

**Database**	**Search strategy**	**# of results**
**Gift**	(“tracing apps”) AND (sovereignty)	10
**Scopus**	“tracing apps” sovereignty	0
**PubMed**	(“tracing apps”) AND (sovereignty)	0
**Google Scholar**	“tracing apps” sovereignty, since 2020	167

The search strategy resulted in a total of 177 publications. I limited the review to articles written in English or German and excluded all papers written in other languages (5). I then excluded all self-published papers, student papers, grey literature, and blog posts (90), before removing the duplicates (12) as well as the articles whose full text I could not access (13). After removing the papers that did not meet the formal requirements, I screened the remaining 57 full-text publications to examine whether they meet the content requirements to include them in my review or not. I included a paper in my final sample if it mentions contact tracing apps in connection to sovereignty. I excluded papers that mention either sovereignty or contact tracing apps (or both) only in the references (8) as well as papers that use sovereignty as part of a proper name (1)—e.g., Sandvik (2020) who talks about “Norway’s sovereignty wealth fund”. I further excluded 20 papers that mention tracing apps and sovereignty separately in two different contexts, with no connection between the two concepts. After removing the papers that did not meet the content requirements (29), 28 papers were included in the final sample.

I then analyzed the final sample with the goal of identifying the interaction between actors described as articulations of “sovereignty.” To do so, I applied the inductive methodology of actor-network-theories—i.e., empirically tracing which social actors appear in a defined area and inductively following how they relate to and interact with each other (Latour, 2005; Latour & Woolgar, 1986)—to the discussions around contact tracing apps. In other words, I put a discourse-analytic twist on the actor-network theory method, and, instead of empirically tracing actors in a social environment, I work out which actors are mentioned in the sample to interact with other actors and articulate their “sovereignty” in the context of contact tracing apps. This allowed me to gain a first and partly disordered insight into the multiple ways in which different actors are said to exercise, claim, or defend their “sovereignty” in the context of contact tracing apps. The next step was to bring order to these various articulations of “sovereignty” by grouping similar articulations together, i.e., articulations by the same actors against the same actors. Finally, I focused on the different groups of “sovereignty” articulations in the context of contact tracing apps and worked out in each case *how* the actors are described to articulate their “sovereignty” against each other, e.g., by exercising power, claiming sovereignty, or threatening the sovereignty of others, as well as how these articulations of “sovereignty” are called. This finally resulted is an ordered overview of the different articulations of “sovereignty” in the context of contact tracing apps that can be found in the sample. This overview is neither systematic nor conclusive—i.e., it does not provide statistical information about the frequency of each articulation and there might also be a good chance that there are other articulations of “sovereignty” in the context of contact tracing apps. But the overview is very well representative for the sample, gives an insight into the most frequently mentioned “sovereignty” articulations in the specific context, and allows a better understanding of sovereignty in the digital.

## Results

The analysis of the publications on sovereignty and contact tracing apps showed that three actors are considered to play a central role: individuals that use contact tracing apps and produce data through contact tracing apps, *(big tech) companies* that produce smartphones and develop the operating systems that the contact tracing apps are running on, and *nations* or *national governments*—I use both terms synonymously—that are centrally involved in the process of developing contact tracing apps, by setting requirements for the apps and enact legal regulations, and that use the data collected for public health purposes. This is in line with the above considerations on sovereignty in the digital (see Sect. 2.3) as well as previous publications on digital sovereignty (see Sect. 6.3) and supports their general theoretical view on central actors of sovereignty in the digital.

These actors are said to be working together to develop, implement and use contact tracing apps in the containment of the COVID-19 pandemic. However, despite their common goal, several actions are identified, by which the actors try to exercise power against each other and to manipulate each other. Such actions are identified by many authors as articulations of “sovereignty” and are attributed to dissents about how contact tracing apps should work and how they should store and analyze their data and who should have control over this data. Each of the three actors is found to exercise their power against each of the other two actors—resulting in a total of six different articulations of “sovereignty” in the context of contact tracing apps, that I will present in this section after describing a central dissent about contact tracing apps in an introductory section.

### Dissent Around the French StopCovid Contact Tracing App

A key reference point often used as *the* example when discussing sovereignty in the context of contact tracing apps is the development of the former French contact tracing app *StopCovid*—today France is using the app *TousAntiCovid* (République Française, 2022) —which worked by exchanging Bluetooth data to collect contact information. In the process of developing this app, the French government favored a centralized approach to contact tracing apps, meaning that the data that the app collects is stored centrally on a French server in anonymized form (Dillet, 2020). Many critics raised concerns about the app’s privacy when following a centralized approach and instead favored a decentralized approach, meaning that the data the app collects is stored only on the smartphone itself. A decentralized approach, the critics argued, can guarantee the users’ privacy better (Lomas, 2020). Despite these concerns and criticism, the French government stuck to its plan to develop *StopCovid* with a centralized approach and emphasized that the app meets high privacy standards (Osborne, 2020).

To complicate matters, third-party apps, in this case from the French contact tracing apps, have one central disadvantage. For privacy reasons, apps on iPhones running Apple’s iOS and on smartphones running Google’s Android operating system—the two smartphone and operating systems manufacturers with the largest market share, together over 99%, in Europe (statcounter, 2022)—can only send Bluetooth signals when the app is opened. As soon as the app is closed, it is prohibited from sending Bluetooth signals at the level of the smartphone’s operating system.

Apple and Google could lift this restriction by releasing the Bluetooth API for the app, which would allow it to send and exchange Bluetooth data in the background even when it is closed. However, in the case of contact tracing apps, Apple and Google agreed to release the required API only under two conditions: first, there must be only one contact tracing app per nation and, second, it must follow a decentralized approach and store the data solely on the users’ smartphones (Scott et al., 2020). Apple and Google stated that the reason for this decision was to protect the privacy of their users (Etherington & Lomas, 2020).

This led to a “standoff” between the French government and its centralized approach and Google and Apple, favoring a decentralized approach (Rosemain & Busvine, 2020). While other governments that faced similar problems—e.g., Germany and its *Corona-Warn-App*—switched their app’s approach to meet Google’s and Apple’s conditions (Busvine & Rinke, 2020) and got access to the Bluetooth API, the French government remained rigid and kept its centralized approach (Lovejoy, 2020). This led to the French *StopCovid* app having a massive flaw: it had to always be opened to send and exchange Bluetooth signals and correctly trace the users’ contacts. This always-being-open, in turn, is not only impractical but also has a negative impact on the smartphone’s battery performance (Montagni et al., 2020).

### Nations → (Big Tech) Companies

In this “standoff “ (Rosemain & Busvine, 2020), both nations and (big tech) companies sought to exert control over contact tracing apps and determine how they should collect and store their users’ data. Some authors recognized these attempts to control contact tracing apps as articulations of sovereignty and use the concept of sovereignty to describe this standoff. For example, Jacob and Lawarée (2020) see France’s stand for their centralized approach as an expression of their “digital sovereignty” that is, in turn, one central part of France’s national AI strategy (Villani et al., 2018).

“This principle of digital sovereignty grants power to the French government to make decisions regarding algorithms and to control the data necessary to carry out its policies.” (Jacob & Lawarée, 2020, p. 13)

Both authors claim that it is crucial for France’s sovereignty in the digital to maintain their app’s centralized approach and not let (big tech) companies influence and control the decisions on how their contact tracing apps should work.

Another reason why it is important that the app follows a centralized approach is given by Altshuler and Hershkovitz (2020). They focus on the app’s functionality and claim that it is crucial for France's public health authorities to have access to the information about “who was in physical proximity to a person with COVID-19 and therefore may be exposed to the virus.” (5) If the public health authorities can access this data, they can counteract the COVID-19 pandemic more effectively. And as this access is only possible for public health authorities if the app’s data is stored centrally, it was important for France, so Altshuler and Hershkovitz (2020), to defend the “app’s sovereignty” (5), i.e., make sure that its data is stored centrally.

Moreover, Altshuler and Hershkovitz (2020) fear that, if the French government had given in to Google’s and Apple’s requirements, these two (big tech) companies might in turn have had access to French citizens’ contact tracing data. This would have threatened their privacy even more than the government and its authorities having access to this data—and might weaken the trust the French citizens have in their government in the long term. Better protection of the citizens’ data would in turn increase their trust in the app (Rowe, 2020, pp. 2–3). This is why, according to the authors, it was crucial that the French government:

“made it an issue of national sovereignty to try to regain trust of citizens who may otherwise fear that their personal and health data may be monetized by tech giants.” (Rowe, 2020, pp. 2–3)

As the above examples show, France’s refusal to give in to the demands of Google and Apple and develop their contact tracing app with a centralized approach is understood as an expression of France’s digital and national sovereignty. This exercise of sovereignty is justified by claiming that a decentralized app enables the public health authorities to work more effectively and at the same time guarantees better data protection (White & van Basshuysen, 2021), which in turn contributes to increasing citizens’ trust in the app. The reference to the welfare of citizens serves to legitimize France’s exercise of sovereignty against (big tech) companies—and fuels calls for more digital sovereignty for nations (Dohse & Vehrke, 2020).

### (Big Tech) Companies → Nations

Conversely, the demands of Google and Apple, as the two paradigmatic (big tech) companies in the context of the above standoff, are also understood as an expression of sovereignty. As Matt Stoller, director of research at a Washington, D.C.-based think tank focused on preventing and reducing the power of (digital) monopolies, notes:

“They [Google and Apple–MT] are exercising sovereign power. It’s just crazy,” that with those two companies “[y]ou have a private government that is making choices over your society instead of democratic governments being able to make those choices.” (Albergotti & Harwell, 2020)

By using their control over necessary APIs as leverage to dictate national “governments how they can collect data from their citizens in the fight against COVID” (Moerel & Timmers, 2021, p. 6) and how their contact tracing apps should store this data, Google and Apple are exercising what in other contexts is called “hardware and software sovereignty” (Aldrich & Karatzogianni, 2020, pp. 2930): a form of sovereignty that is based on the actor having control over key IT-components and digital infrastructure.

By exercising their hard- and software sovereignty as just described, Google and Apple effectively threaten and limit the sovereignty of nations and their governments (Floridi, 2020; Kliestik & Nesterova, 2021). Likewise, France stated that Google’s and Apple’s refusal to release their API, would be “an attack on their sovereignty” (Veale, 2020, p. 38). Yet, as Knodel (2021) criticizes, neither France nor other nations used “their sovereign and regulatory powers to limit tech companies” (Knodel, 2021, p. 47). Instead, as Veale (2020) observes, they showed:

“little appetite to attempt to rectify this situation with the legal obligations that sovereign states have at their disposal; instead reifying the view of tech giants as state-like themselves, diplomatic interlocutors rather than firms operating under national law. Sovereignty was mourned before any of its traditional tools were even reached for.” (Veale, 2020, p. 38)

Hence, the above standoff proves to be a „power play between sovereign states and the corporations, in which sovereign states had little say.” (Sharon, 2020) But when states have only little say in this standoff and (big tech) companies dictate them how to design their contact tracing apps (Sharon, 2020), these companies gradually withdraw sovereignty from nations (French et al., 2020). As a consequence, sovereignty is gradually shifting from countries to the (big tech) companies.

These remarks show how (big tech) companies have their own form of hardware and software sovereignty and use it to exercise control on how nations should develop their contact tracing apps. By exercising their sovereignty, (big tech) companies threaten the (digital) sovereignty of nations (Mann et al., 2020). Where the latter cannot defend themselves against this threat, they increasingly lose their sovereignty to (big tech) companies. If calls for more state sovereignty are subsequently made (Dohse & Vehrke, 2020), this only points to an existing deficit and shows how eminent the threat to state sovereignty posed by the hardware and software sovereignty of the (big tech) companies is.

### Nations → Individuals

Some authors use the concept of sovereignty to describe how nations introduce contact tracing apps into their populations and encourage or require their citizens to use them. Following Foucault’s considerations on biopolitics, they describe this state action as an expression of so-called “biopolitical sovereignty” (Makarychev & Wishnick, 2022, p. 17).

Foucault develops his concept of biopolitics centrally in his Lectures at the College de France 1975–1976 *The Society must be defended* (Foucault, 2003) and 1978–1979 *The birth of biopolitics* (Foucault, 2004). In these lectures, he shows how the idea that society must be defended leads to a change in how sovereign power is exercised. In the Middle Ages, the sovereign kept out of the lives of the population as much as possible, intervening only to punish them in cases of disobedience. In the Modern era, the sovereign began to increasingly intervene in the lives of the population in order to regulate how they should live. The goal of these regulations was to protect and increase the population. This changed the form of sovereign exercise of power: from a medieval “let live and make die” to a biopolitical “make live and let die” (Foucault, 2003, pp. 239–264). On the one hand, such a Foucauldian biopolitics serves to protect and prolong the life of the population. On the other hand, it massively regulates this life and restricts the freedom of individuals in the population (Siisiäinen, 2017). Therein lies “the ambivalence of biopolitics” (Bazzicalupo, 2006).

This biopolitical ambivalence of protecting the population by regulating their lives is also recognized by some authors in the case of contact tracing apps. By monitoring the contacts and infection statuses of their users, contact tracing apps can contribute to protecting them from infections on the one hand. On the other hand, nations can use contact tracing apps to deeply intervene in the lives of their citizen and exercise a kind of regulation. This kind of regulation can be observed where contact tracing apps are used to give instructions to their users—for example, to get tested for infection as soon as possible or to quarantine themselves—and where they are used, if necessary, to check whether their users follow these instructions—some contact tracing apps also have a function that checks compliance with the quarantine (Brunner et al., 2020). In extreme cases, according to Duncker (2020), this might lead to contact tracing apps “structuring the life and death conditions of feature groups.”

But even where contact tracing apps do not give instructions to their users or monitor their compliance, but only collect and analyze their data, Galloway (2020) claims that they still deeply intervene in their users’ life. For this data, Galloway states with reference to Haraway’s concept of the ‘self as cyborg’ (Haraway, 1991), is an essential part of the human being—and controlling this data via contact tracing apps allows nations to express a form of biopolitical sovereignty. Referring to the Australian contact tracing app *COVIDSafe*, she writes:

“Regardless of legislative prohibition on coercion regarding COVIDSafe, if the app and its information are integral to one’s body, then the app is an exercise of biopower. Given the broader social context of the app (illustrated above), its adoption represents the socialisation of biopower including the exercise of sovereign power.” (Galloway, 2020, p. 166)

Beyond their biopolitical ambivalence, some authors are concerned that contact tracing apps will serve as a kind of “Trojan Horse” (Davis, 2020) to establish a long-term biopolitical sovereignty regime, i.e., to secure physical territorial borders (Yang et al., 2020), to monitor the citizens in a system of “panoptic surveillance” (Keshet, 2020), and to control them through biopolitical surveillance and disciplinary measures. In the course of this scenario, contact tracing apps are viewed as means to normalize a direct exercise of sovereignty and surveillance among the population (Lee & Lee, 2020; Švedkauskas & Maati, 2020) and to pave the way for further biopolitical surveillance and power instruments:

“Today, as efforts are already underway to introduce new instruments of biopolitical governance in response to COVID-19 (such as immunity passports, thermal cameras, geolocation devices, and contact tracing apps), it is clear that some see this current pandemic as an opportunity to ensure that forms of disciplinary power and bio-surveillance permeate even deeper into the management of bodies, households, populations, and the microbiome.” (Simpson, 2021)

Where biopolitical measures are not withdrawn after the end of the pandemic, some authors fear, but remain intact as instruments of the exercise of sovereignty—contact tracing apps being only one and a relatively mild tool—liberal orders are at risk of turning into illiberal orders (Barnett, 2020) and fostering a “Rise of the Digital Surveillance State” (Švedkauskas & Maati, 2020).

These remarks show how the use of contact tracing apps can be interpreted as a form of biopolitical exercise of sovereignty. Where national governments use contact tracing apps to counter the COVID-19 pandemic, they protect their populations on the one hand. On the other hand, this protection is bought by surveillance of the population, invasive intervention in and, in part, regulation and restriction of their lives. In this way, national governments are exercising biopolitical sovereignty against their citizens—and there are concerns about whether this kind of exercise of sovereignty will be reversed after the pandemic ends or further expanded into a biopolitical sovereignty regime.

### Big Tech → Individuals

Where nations exercise biopolitical sovereignty, they harm the sovereignty of their citizens, i.e., limit their ability to make their own decisions free from coercion or manipulation. However, as various authors note, not only nations but also large technology companies exercise sovereignty and thereby threaten the autonomy of individuals—with contact tracing apps playing a crucial role in this as well, according to some authors.

Central to these authors’ considerations are Zuboff’s (2019) reflections on surveillance capitalism. As Zuboff (2019) shows, (big tech) companies, especially digital platforms, monitor their users around the clock and collect their data up to that point that they know, as Grey puts it in his reflections on Zuboff, “more about us than we know about ourselves” (Gray, 2019). This data can be used, sometimes by third parties, to influence the decisions of individuals—e.g., when it comes to personalized advertising (Jackson, 2021) or, as for example the Cambridge Analytica scandal surrounding the 2016 Trump election and Brexit illustriously illustrates (Wylie, 2019), in political contexts. By collecting their users’ data, (big tech) companies can compromise their users’ autonomy, establishing themselves as “a new kind of sovereign power.” (Zuboff, 2015, p. 86).

Several authors pick up on these reflections and show how contact tracing apps promote these surveillance capitalistic dynamics and contribute to strengthening (big tech) companies’ “new kind of sovereign power” (Zuboff, 2015, p. 86) and threatening the autonomy of individuals—although rarely explicitly mentioning the term sovereignty. For example, Whitehead (2020) warns that “surveillance capitalism has a historical track record of exploiting crises” and that (big tech) companies could see the COVID-19 pandemic and especially the use of contact tracing apps as an opportunity to gain even more personal data and to expand their power. In an interview with *Computer Weekly*, Zuboff expresses similar concerns:

“For a company like Google, creating contact-tracing applications in partnership with Apple or the many other ways in which it wants to lend its resources for disease tracking and containment, the great likelihood is that these become institutionalised as new supply chains for Google[.]” (Klovig Skelton, 2020b)

When (big tech) companies gain access to large amounts of personal data through contact tracing apps, this enables them to monitor their users even more extensively (Garrett, 2021). This is how, Whitehead (2020) notes, contact tracing apps “could contribute to the growing power and influence of surveillance capitalism over our lives.” These concerns are shared by Boddington (2021), as well as Keilitz (2020), who both warn that the use of contact tracing apps can contribute to increasing the sovereignty of (big tech) companies. These concerns are further heightened by the fact that many contact tracing apps don't “have any publicly stated anonymity measures” (Woodhams, 2021) and that contact tracing data can be repurposed for other purposes (Klovig Skelton, 2020a).

These remarks show how (big tech) companies, by collecting vast amounts of data about individuals and thereby gaining the ability to influence their decisions, threaten the autonomy of individuals and gain sovereignty of their own. By participating in this extensive data collection, contact tracing apps contribute to the emergence of new big tech sovereigns and to their threat to individual autonomy.

### Individuals → Nations and (Big Tech) Companies

As we have seen, the autonomy of individuals—and, by analogy, the autonomy of groups of individuals (Hyland-Wood et al., 2021; Manning & Walton, 2021) —in the context of contact tracing apps is threatened from two sides. On one side, nations exercise a form of biopolitical sovereignty through the implementation of contact tracing apps. On the other side, (big tech) companies permanently collect data of individuals—among others through contact tracing apps—and establish themselves as a “new kind of sovereign power” (Zuboff, 2015, p. 86). And the more access nations or (big tech) companies have to individual data, the stronger their sovereignty becomes and the more invasively they can intervene in the lives of individuals and threaten their autonomy.

The intuitive idea of protecting one’s autonomy by not producing any data has the disadvantage that one could then no longer use contact tracing apps. The individual is thus faced with a “privacy paradox” (Rowe, 2020)—either to protect one’s autonomy and forego the benefits of contact tracing apps (Bengio et al., 2021) or to enjoy the benefits of these apps, but to disclose one’s own data in return. The solution of preserving one’s own autonomy through data minimization (Fathauer, 2020) likewise works only to a limited extent. Firstly, contact tracing apps need a minimum amount of data, which cannot be undercut, and secondly, even small amounts of data can increase the sovereignty of nations and (big tech) companies and can contribute to undermining one’s own autonomy.

A creative solution that allows individuals to share their data so that they can effectively use contact tracing apps, while allowing them to maintain control over their data and decide which actors may use it, for what purpose (and potentially for how long), is the concept of “data sovereignty” (German Ethics Council, 2018). In the context of contact tracing apps, such data sovereignty has been frequently called for (European Parliament, 2020; Floridi, 2020; Grantz et al., 2020; Mauro, 2020)—though, as Micheli et al. (2020) and Grantz et al. (2020) criticize, it has not been adequately implemented. The challenge in implementing data sovereignty is that there is a fundamental asymmetry of power between individuals, who want to have sovereign control over their data, and nations or (big tech) companies, both of which are interested in accessing the individual’s data. As a consequence, it is difficult for individuals to establish themselves as data sovereigns—to do so, they would need advocates.

At this point, nations and (big tech) companies step up as advocates for individuals and their data sovereignty. In the case of the *StopCovid* app described above, Apple and Google are using their own hardware and software sovereignty and try to push the French government to switch from a centralized to a decentralized approach. The reason given by the two (big tech) companies for this move is, as mentioned above, that the app users’ data and their privacy would be better protected against direct access by the state and its institutions. This results in users being able to control who accesses and uses their data—i.e., in an increase in their individual data sovereignty and, conversely, in a limitation of the biopolitical sovereignty of nations. Thus, (big tech) companies act as advocates of individual data sovereignty against biopolitical exercises of sovereignty by nations. In turn, nations act as advocates of individual data sovereignty by enacting legal regulations that allow the collection and analysis of individual data only with the consent of individuals. Thus, nations use their legislative sovereignty to limit the sovereign power of surveillance-capitalist tech companies—and to promote the data sovereignty of their citizens. In the context of the digital fight against the COVID-19 pandemic, the European *GDPR* played a major role and set the legal framework that tech companies had to comply with (Bradford et al., 2020).

These remarks show how individuals claim sovereign control over their contact tracing app data in order to protect themselves from the sovereignty exercises of nations and (big tech) companies. Since it is difficult for individuals to establish themselves as sovereigns over their data, they need advocates to obtain data sovereignty. When nations or (big tech) companies, through exercising their own hardware, software, or legislative sovereignty, limit third parties’ exercise of sovereignty over individuals, they act, sometimes without realizing it, as such advocates—and open up a space where individuals can gain data sovereignty.

## Discussion

The *Results* showed that actors play a central role in the discussions on contact tracing apps: *individuals* that use contact tracing apps and produce data, *(big tech) companies* that produce smartphones and develop the operating systems that the contact tracing apps are running on, and *nations* that are centrally involved in the process of developing contact tracing apps, by setting requirements for the apps and enact legal regulations, and that use the data collected for public health purposes. These three actors are said to exercise power *against* and *with* each other, that are often described using the term “sovereignty.” In this section, I will first reflect on how the term “sovereignty” is used in the sample and how this relates to the considerations above about the concept of sovereignty. Then, I will weave together the various articulations of sovereignty in order to get an impression of the complex network that is sovereignty in the context of contact tracing apps. Following this, the results will be brought into conversation with other authors’ conceptions of “digital sovereignty” to identify commonalities and differences between them and to find out whether the findings from the particular context can be generalized and allow conclusions to be drawn for sovereignty in the digital in general. Finally, I will address the limitations of this study.

### The Use of the Term Sovereignty in the Sample

The results show how the exercise of power by the three actors in the context of contact tracing apps is described by different actors with the help of the term “sovereignty.” It is striking that the exercise of power is often described as “sovereignty” and both concepts are widely used synonymously. Against the background of the considerations from chapter 2, however, this use of the term proves to be difficult. For sovereignty was described there as the *supreme authority within a territory* (Philpott, 2003), i.e., the authority that decides which forms of power are legitimate and which are not. While there is indeed a close connection between power, authority, and sovereignty, not every exercise of power, nor every exercise of authority can be described as sovereignty. Rather, it has been shown that in digital contexts there is no sovereign at all, but only an ongoing “struggle” (Pohle, 2021) for sovereignty.

In order neither to dilute the concept of sovereignty and fall behind the considerations in chapter 2 by simply adopting the use of terms from the sample, nor to reject the term “sovereignty” altogether, I propose: When the sample speaks of an actor “exercising sovereignty,” this is most likely to be understood as the actor exercising power to increase its authority and establish itself as sovereign as well as, conversely, to either undermine or underpin the authority of other actors, thereby weakening or strengthening their claims to sovereignty. Ergo, these are not *exercises of sovereignty*, but *exercises of power associated with different claims to sovereignty*. In order to designate the latter and to position myself between the use of the term in the sample and the above conceptual considerations, I will speak below—and have before—of “articulations of sovereignty.”

### Weaving the Various Articulations of Sovereignty into One Complex Network

As the results show, three actors exercise power with and against each other in order to claim sovereignty or to weaken or strengthen the sovereignty claims of the other actors. In total, there are six such articulations of sovereignty.

1. (Big tech) companies → Nations: (big tech) companies *articulate* their hardware and software sovereignty against nations to dictate them how their contact tracing app should store its data. In doing so, (big tech) companies *threaten* the digital sovereignty of nations and open up space for individuals to claim their data sovereignty.
2. Nations → (Big tech) companies: National governments feel their digital sovereignty is *threatened* by (big tech) companies. Where they resist the demands of the latter, this is seen as an expression of their digital sovereignty. In addition, nations’ governments are *articulating* their legislative sovereignty to dictate (big tech) companies how they may collect data. In doing so, nations are *opening up* a space for individuals’ data sovereignty.
3. Nations → Individuals: Nations use contact tracing apps to *articulate* what is called “biopolitical sovereignty” (Makarychev & Wishnick, 2022, p. 17) over their citizens, i.e., to protect their lives by regulating them. The intention to protect the population legitimizes this exercise of sovereignty. Where the protection of the citizens is no longer intended, but other interests become central, this legitimacy disappears.
4. (Big tech) companies → Individuals: In surveillance capitalism, (big tech) companies permanently collect data from individuals and use it, as “a new kind of sovereign power” (Zuboff, 2015, p. 86), to influence individuals in their decisions—*threatening* their individual sovereignty. Contact tracing apps facilitate this sovereign power process by contributing to data collection and analysis.
5. Individuals → Nations *and* (big tech) companies: To prevent nations and (big tech) companies from exercising their sovereignty over them and thus *threatening* their autonomy, individuals *claim* data sovereignty, i.e., control over their data. But since they have less power than the other actors, they cannot achieve data sovereignty on their own. In the context of contact tracing apps, (big tech) companies and nations act as advocates where, by *exercising* their own sovereignty, they limit how much the respective third parties exercise power over individuals—thus *opening up* space for individual data sovereignty.

**Fig. 1:**
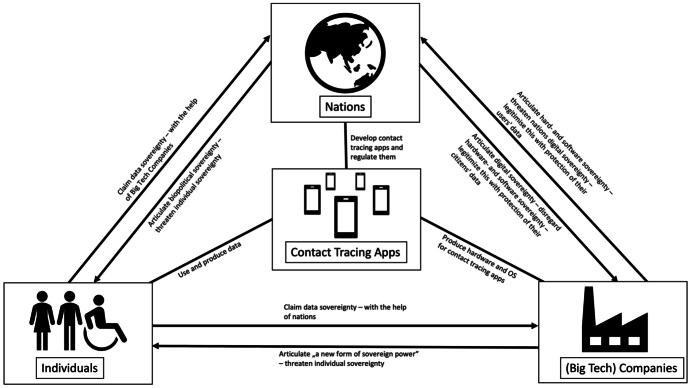
Illustration of the different ways in which the three different actors*—*nations, (big tech) companies, and individuals*—*articulate their sovereignty, i.e., exercise power with or against each other in order to claim sovereignty and to prevent or enable the sovereignty claims of other actors, in the context of digital contact tracing apps (created by the author)

These six articulations of sovereignty can be put together to give a sense of how the various actors exercise power to claim sovereignty or to weaken or strengthen the claims of others. The result is a network of sovereignty articulations that spans between the three actors. As shown in chapter 2, sovereignty in the digital realm can be understood as a network of different exercises of power. Similarly, sovereignty in the context of contact tracing apps can be understood as a complex network of three actors, nations, (big tech) companies, and individuals, who exercise their power in different ways, including through software, hardware, or laws. And by exercising their power, they claim sovereignty in the context of these apps, strengthen or weaken the sovereignty claims of the other actors. These complex entanglements of sovereignty articulations can be illustrated (see Fig. 1)—and as a whole represent the structure of sovereignty in the context of contact tracing apps.

### Conceptional Discussions

Next, I would like to discuss three central findings in more detail and bring them into conversation with existing considerations on “digital sovereignty. “The aim of these conceptual discussions is to identify similarities and differences between existing considerations of “digital sovereignty” and the observations made above—and to see whether the insights gained about sovereignty in the particular context of contact tracing apps can be applied for the digital in general. The three points I will discuss in more detail are the three central actors of sovereignty in the context of contact tracing apps, the fact that sovereignty in digital depends on various analog as well as digital factors, and the way actors exercise their power with and against each other.

First, the results above have shown that three actors play a central role in the context of contact tracing apps and struggle for sovereignty: nations, (big tech) companies, and individuals. In a similar vein, Julia Pohle assumes that “digital sovereignty” takes place in three dimensions and includes state, economic, and individual actors (Pohle & Thiel, 2020, 2021; Pohle, 2020a, b, 2021). Similarly, Luciano Floridi distinguishes between political actors (France, Germany, UK, China, EU), economic actors (Huawei, Amazon, Apple, Google, Facebook, Microsoft), and individuals (citizens, private citizens) when thinking about “digital sovereignty” (Floridi, 2020). The distinction between state, economic, and individual actors found in Pohle and Floridi shows great similarities to the distinction between nations, (big tech) companies, and individuals identified in the context of contact tracing apps.

Second, the results above have shown that both analog and digital factors, e.g., analog infrastructures and hardware as well as digital data and software, are important to achieve and maintain sovereignty in the digital—an idea that is also reflected in various considerations on “digital sovereignty” by others. For example, in their impulse paper on *Digital Sovereignty*, Kagermann et al. (2021) develop a “technology layer model” and list various factors—including access to raw materials and intermediate products, but also digital “data spaces” —that are necessary to achieve and maintain “digital sovereignty.” Several other publications, that focus on analyzing “digital sovereignty strategies” (Baischew et al., 2020) of Russia, China, or the European Union, additionally confirm that digital as well as analogue factors are needed to establish and secure sovereignty in the digital. From secure access to rare earths and raw materials (Metakides, 2022), reliable and secure infrastructure (Braud et al., 2021; Griffiths, 2021; Ristolainen, 2017), to legal regulations, e.g., on data protection (Roberts et al., 2021), data localization (Ermoshina & Musiani, 2017), or the use of VPN services (Nguyen-Thu, 2018), and fixed boundaries in the digital (Kukkola & Ristolainen, 2018). This observation that both analog and digital factors are important for sovereignty in the digital is a second commonality between previous considerations about “digital sovereignty” and the above findings.

Third, the results and, following them, the discussion, have shown that sovereignty in the context of contact tracing apps can be understood as a network of different articulations of sovereignty. By exercising power, the actors claim sovereignty for themselves and aim to *either* weaken *or* strengthen the sovereignty claims of other actors. The latter, in particular, proves interesting. Although other authors also understand “digital sovereignty” as a network of power exercises, e.g., Floridi in *The Fight for Digital Sovereignty* (Floridi, 2020), they largely assume that the actors exercise their power mainly *against* each other. That is, that they seek sovereignty in the digital by *weakening* the sovereignty claims of others. Contrary to these assumptions, the above Sect. 5 show that actors also exercise their power on behalf of each other. For example, where (big tech) companies articulate their hardware and software sovereignty to pressure nations to refrain from a centralized model of contact tracing apps—thus supporting individuals’ claims for “data sovereignty.” Or when nations enact laws to protect their citizens’ data from (big tech) companies—thereby strengthening the latter’s “data sovereignty.” Admittedly, one can also object in these examples that actors can only support the claims of other actors by exercising their power against third parties and weakening their sovereignty claims. But exercising power *against* third parties and the *weakening* of their sovereignty claims is only one side of the coin. The other side is the positive aspect, exercising power for others and strengthening their claims. The fact that these results take both sides of the coin into account and do not only focus on the negative exercises of power and the actors mutually weakening their sovereignty claims is the central difference between these and previous considerations on “digital sovereignty.”

In summary, there are several similarities between the results on sovereignty in the context of contact tracing apps and existing considerations on “digital sovereignty.” Both identify similar actors—nations, big tech companies, and individuals, or state, economic, and individual actors—and assume that digital as well as analog factors must be considered to achieve and maintain sovereignty in the digital. And while both understand sovereignty in the digital as a network of different exercises of power, a key difference is how these are perceived. Whether the focus is on the *negative* exercises of power that are directed *against* other actors and aim to *weaken* their claims to sovereignty. Or whether attention is also paid to the *positive* exercises of power *on behalf* of others, aimed at *strengthening* their sovereignty claims. At this point, a brief recourse to chapter 2.2 is helpful. There it was shown that sovereigns do not strengthen their position exclusively by exercising their power *against* others and weakening their sovereignty. Rather, it can be advantageous for sovereigns to cooperate with other sovereigns and strengthen their sovereignty. Against this background, it seems reasonable to always consider both aspects in the digital as well and to look at how actors exercise their power *against* each other and against each other’s sovereignty claims, but how they also exercise their power *on behalf* of each other—and strengthen their sovereignty through both articulations.

### Limitations

There are two central limitations to my results. First, the article asks how sovereignty can be understood in the context of digital contact tracing apps—but then only partially incorporates the stakeholders’ perspectives and statements, and focuses predominantly on the academic discourse around sovereignty and contact tracing apps. As such, it primarily provides answers to the question as to how sovereignty is understood *in the discussions* around contact tracing apps. Although methodically not absolutely precise—additional analyses of stakeholder perspectives would have been needed—the results are not completely unusable either. Rather, as outlined in the *Methods*, it can even be beneficial to focus on the discussions around these apps, and, as the Sect. 6 shows, this approach also yields productive results and provides productive input for understanding sovereignty in the digital.

Second, the article analyzes sovereignty exclusively in the particular context of contact tracing apps, but then in the Sect. 6 attempts to draw conclusions from this about sovereignty in the digital in general. Justified doubts can be raised against this approach and the objection can be made that solely pointing out similarities does not methodologically legitimize deriving general results (sovereignty in the digital) from a specific context (sovereignty in the context of contact tracing apps). Despite these objections, I am convinced that sovereignty in the context of contact tracing apps is a paradigmatic example of sovereignty in the digital and allows to at least draw conclusions from the former to the latter.

Last but not least, my research is kept entirely descriptive and refrains from normative judgments. It uses the example of contact tracing apps to analyze how different actors articulate sovereignty in the digital against each other. Normative questions, such as whether all articulations of sovereignty presented above are legitimate, whether there are illegitimate articulations of sovereignty in the digital, or which articulations are better or worse, have been consistently excluded. As presented in the Sect. 6, some authors have suggested that articulations of sovereignty are legitimate as long as they contribute to the protection, health, or sovereignty of individuals (Davis, 2020), and that they become illegitimate once the interests of individuals are no longer the focus (Keshet, 2020; Yang et al., 2020). There are, accordingly, already a number of publications that address normative questions about the legitimacy or illegitimacy of digital sovereignty articulations (Glasze et al., 2022a; Roberts et al., 2021; Rosengrün, 2022; Schneider, 2020)—but it might be worth revisiting them in light of these newfound insights into the complexity of sovereignty in the digital.

## Conclusion

Against the background of the observation that sovereignty in the digital is a complex phenomenon, the aim of this paper was to explore the structure of digital sovereignty using one particular example. Digital contact tracing apps were chosen because they are a currently relevant technology and illustrate well the structures of sovereignty in the digital—with different actors involved seeking to enforce their different ideas by exercising power. The research question of the paper was therefore: how can we understand digital sovereignty in the context of contact tracing apps—and what conclusions does this allow about the structure of sovereignty in the digital?

Using a narrative review for which I applied the inductive method of actor-network to the discussions around contact tracing apps, I analyzed a representative sample of discussions around contact tracing apps. I asked how various authors use the concept of sovereignty to describe how different actors interact with each other in the context of contact tracing apps. As a result, it was shown that there are three central actors—nations, (big tech) companies, and individuals—that *articulate sovereignty*. That is, they exercise power against and on behalf of each other to claim sovereignty and to weaken or strengthen the sovereignty claims of other actors. With each actor exercising power against every other actor, there are a total of six different articulations of sovereignty in this context: (big tech) companies against nations, nations against (big tech) companies, nations against individuals, individuals against nations, (big tech) companies against individuals, and individuals against (big tech) companies. As a result, I conclude that sovereignty in the context of contact tracing apps can be understood as a network of mutually conditional and restrictive exercises of power each aiming for sovereignty, which is spun between individuals, nations, and (big tech) companies.

As I have shown in the discussions, sovereignty in the context of contact-tracing apps has many similarities—in terms of actors, structure, and the relationship between various analog and digital means—to existing considerations of “digital sovereignty.” This allows us to draw conclusions about sovereignty in the digital more generally from this particular context. The former can be understood as a complex network of political, economic and individual actors who exercise power to claim or achieve sovereignty. Each of these exercises of power can be directed against the sovereignty claims of other actors, weakening or even preventing them—but it can also counter them positively and strengthen them. In conclusion, sovereignty in the digital can therefore be understood as a network of different actors who exercise different forms of power *with* or *against* each other, mutually *strengthening* or *weakening* each other’s sovereignty claims.

This conclusion can not only help inform future discussions about contact tracing apps and provide participants with a better understanding of sovereignty in this particular context, but it also contributes to a more comprehensive understanding of the structure of sovereignty in the digital as a whole and can improve discussions about it.

## Data Availability

The list of publications in the final sample is available upon request from the corresponding author.

## Footnotes

1 Since the term “digital sovereignty” is discussed so much and is charged with so much meaning, I will instead speak of “sovereignty in the digital.” I will use the term “digital sovereignty” only when referring to other authors who use it.
